# A Paradigm Shift in End-of-Life Membrane Recycling: From Conventional to Emerging Techniques

**DOI:** 10.3390/membranes15120350

**Published:** 2025-11-23

**Authors:** Noman Khalid Khanzada, Yazan Ibrahim, Muzamil Khatri, Mohamed Khayet, Nidal Hilal

**Affiliations:** 1 NYUAD Water Research Center, New York University Abu Dhabi, Abu Dhabi P.O. Box 129188, United Arab Emirates; 2 Department of Structure of Matter, Thermal Physics and Electronics, Faculty of Physics, University Complutense of Madrid, Avda. Complutense s/n, 28040 Madrid, Spain; khayetm@fis.ucm.es; 3 Madrid Institute for Advanced Studies of Water (IMDEA Water Institute), Avda. Punto Com N° 2, Alcalá de Henares, 28805 Madrid, Spain; 4 NYUAD Research Institute, New York University Abu Dhabi, Abu Dhabi P.O. Box 129188, United Arab Emirates

**Keywords:** sustainable manufacturing, recyclable membranes, End-of-Life (EoL) membrane, circularity in membrane technology

## Abstract

The conventional linear life cycle of membrane materials, spanning fabrication, use, and disposal through landfilling or incineration poses serious sustainability challenges. The environmental burden associated with both the production of new membranes and the disposal of end-of-life (EoL) modules is considerable, further intensified by the reliance on fossil fuel-derived polymers, toxic solvents, and resource-intensive manufacturing processes. These challenges underscore the urgent need to integrate sustainability principles across the entire membrane life cycle, from raw material selection to reuse and regeneration. Emerging approaches such as membrane regeneration using recyclable polymers based on covalent adaptable networks (CANs) have introduced a new paradigm of closed-loop design, enabling complete depolymerization and reformation. In parallel, more conventional strategies, including the valorization of recycled plastic waste and the upcycling or downcycling of EoL membranes, offer practical routes toward a circular membrane economy. In this review, we consolidate current advances in membrane recycling, critically evaluate their practical constraints, and delineate the technical and environmental challenges that must be addressed for broader implementation. The insights presented here aim to guide the development of next-generation circular membrane technologies that harmonize sustainability with performance.

## 1. Introduction

The global concern regarding water scarcity has been increasingly prominent due to its pivotal function in upholding environmental sustainability and ecological systems [1]. In the contemporary era characterized by population, urbanization, and industrial escalation, performing desalination and reclaiming wastewater via sophisticated approaches are the only pathways to combat the emerging water scarcity challenge and ensure sustainability in humanity’s hinges [2,3].

For the past few decades, membrane technology has been demonstrating a promising role in attaining this goal [4,5]. Unlike conventional treatment methods, membrane technology offers numerous advantages, including enhanced and consistent quality of treated water, compact and robust design, cost-effective operation, and more [6]. Depending upon the morphological structure, separation efficiency, and the principle of operation, membrane technology is classified into different types [7,8]. Reverse osmosis (RO), nanofiltration (NF), ultrafiltration (UF), and microfiltration (MF) are well-known classifications of membrane-based processes that are considered pressure-driven and are widely implemented at the industrial level [8,9,10]. In fact, the deployment of these methods has been experiencing perpetual growth. Other membrane-based processes, such as forward osmosis (FO), pervaporation (PV), membrane distillation (MD), and electrodialysis (ED) that utilizes ion-exchange membrane (IEM), are also now being implemented, although they are still in the nascent stage for being utilized at a larger scale for various applications [11,12,13,14].

The fabrication of membranes used in these processes predominantly relies on polymers synthesized from non-renewable, fossil-based sources [15]. Common materials include polysulfone (PSf), polypropylene (PP), poly(vinylidene fluoride) (PVDF), polyethylene (PE) poly(ether sulfone) (PES), polyamide (PA), polyacrylonitrile (PAN), poly(ethylene terephthalate) (PET), and polystyrene (PS), among others [16,17,18,19,20,21,22]. While these polymers have been favored for their mechanical robustness, chemical stability, and processability, they are non-biodegradable and inherently persistent in the environment, posing long-term ecological and health risks. Their production, use, and eventual disposal contribute significantly to environmental degradation, particularly when handled improperly. Membrane fabrication often employs highly toxic organic solvents such as 1-methyl-2-pyrrolidone (NMP), N,N-dimethylformamide (DMF), and N,N-dimethylacetamide (DMAc), all of which are classified as Substances of Very High Concern (SVHC) due to their reproductive toxicity and persistence [23]. These solvents present occupational hazards during production, and their utilization in fabricating the membranes and improper handling lead to their discharge into the environment [24].

Given that membrane modules are now being produced at massive industrial scales, the combined environmental burden arising from the synthesis, use, and end-of-life (EoL) management of these fossil-derived polymers and toxic solvents is substantial [25]. Compounding this issue, even high-performing membranes inevitably reach the end of their service life due to fouling accumulation, chemical-induced degradation, and mechanical defects in the polymer matrix [26,27]. Although periodic cleaning and chemical dosing can temporarily restore permeability and selectivity, long-term operation leads to irreversible deterioration [28]. Consequently, deteriorated membranes are routinely discarded and replaced with new elements to maintain required effluent quality standards, further aggravating the cycle of material consumption and waste generation.

In 2012, Lawler et al. estimated the potential waste generation of 12,000 tons/year from the discarded EoL membrane modules by the year 2015 [29]. A study published a decade later, in 2022, reported an annual disposal of one million modules corresponding to 16,700 tons (i.e., 37,500 m^3^ of waste) [30]. The current and projected number for their disposal is anticipated to be even higher due to the carried out and ongoing rapid expansions of desalination facilities, which are being governed by the accelerating water demand. The disposal of these membranes typically adheres to the waste management legislation of individual countries, which prescribes their incineration or landfill disposal. Nevertheless, the detrimental environmental repercussions linked to both endure. For example, the degradation of a commonly used spiral wound membrane (SWM) module in a landfill requires several years, whereas inadequate waste management and control during the incineration carried out for energy recovery can lead to the emissions of greenhouse gases and other harmful/toxic byproducts [31]. In addition, the utilization of resources for fabricating these membrane modules is alarming as it does not comply with the circular economy and environmental sustainability concept. These factors emphasize the crucial need for circularity inclusion and for developing sustainable and environmentally friendly methods for manufacturing membranes [20,32].

In view of the escalating environmental footprint associated with membrane fabrication, use, and disposal, attention has recently shifted toward strategies that align membrane technology with the principles of the circular economy and resource recovery. Among these, recycling and repurposing of EoL membranes have emerged as potential pathways to mitigate waste generation, reduce reliance on virgin fossil-based polymers, and extend the value chain of existing materials [33,34,35,36]. However, despite notable progress in converting spent RO membranes into functional membranes, several barriers still impede large-scale adoption. Questions remain regarding the long-term performance, cost-effectiveness, scalability, and sustainability of such recycled products compared with conventional alternatives. 

This review systematically compiles and critically evaluates existing and emerging end-of-life (EoL) membrane recycling strategies. It compares conventional and advanced approaches in terms of technical feasibility, environmental impact, and scalability, while identifying their key challenges and potential synergies. Through this framework, the review aims to determine whether recycling can realistically transition membrane technology toward a circular and environmentally responsible paradigm or if alternative innovations are required to achieve that goal.

## 2. Recyclability in Membrane Technology

Recyclability in membrane technology refers to the reutilization of EoL or used membranes for similar or up-/down-graded separation processes, as well as the recovery and reuse of valuable polymeric materials for the fabrication of new membranes [37]. It also encompasses the repurposing of plastic waste as a feedstock in membrane manufacturing, thereby extending the life cycle of polymeric resources within the separation industry.

**Figure 1:**
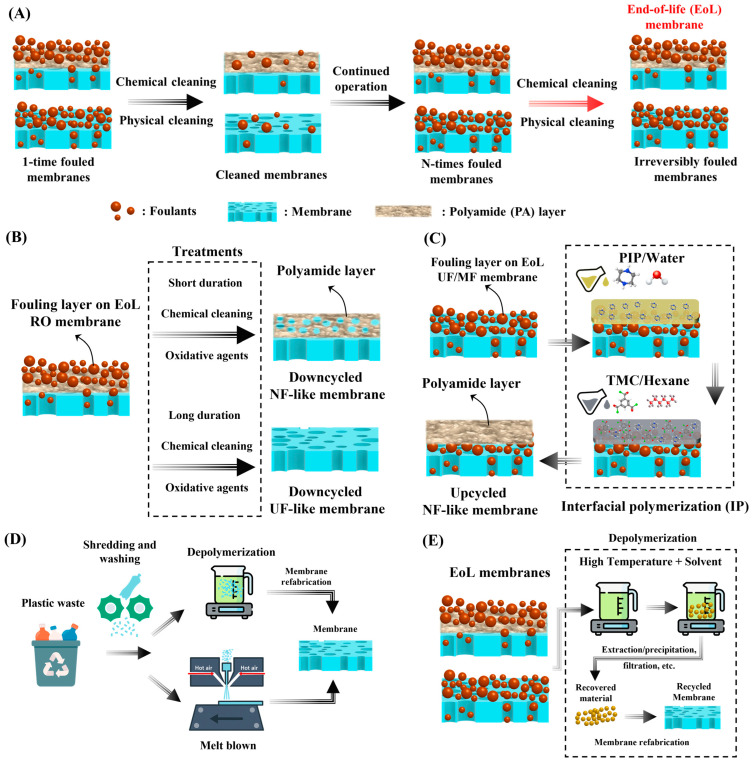
(**A**) Illustration of a membrane lifetime operation cycle. (**B**,**C**) Down- and upcycling of fouled membranes. (**D**) Repurposing of plastic waste in membrane fabrication. (**E**) Emerging depolymerization-based recovery method for membrane recycling.

As illustrated in Figure 1A, a typical membrane undergoes multiple stages during its operational lifetime. Following installation and initial operation, membranes gradually accumulate organic, inorganic, and biological foulants, which are periodically removed through chemical or physical cleaning. However, a fraction of these contaminants remains irreversibly attached to the surface or within the pores. After repeated cycles of fouling and cleaning, the membrane’s performance irreversibly declines reaching the EoL stage. For RO and NF membranes, this typically occurs after 5–7 years of service [14,38,39], whereas for UF and MF membranes, this occurs every 5–10 years [38,40]. At this stage membranes are usually discarded rather than recovered. This linear “use-and-dispose” model underscores the growing need for recyclability-oriented approaches, which aim to extend material utility and minimize waste generation.

Recent strategies to address this challenge can be broadly categorized into three main directions, illustrated in Figure 1B–E. The first involves up- and downcycling of fouled membranes, where membranes are either converted to higher-performance configurations (e.g., modifying fouled UF/MF membranes into NF or RO) or repurposed for less demanding applications (e.g., degrading RO membranes into NF/UF). The second focuses on reusing post-consumer plastic waste in membrane fabrication, thereby transforming discarded polymers such as polyethylene, polypropylene, or PET into functional separation materials. The third and most recent direction explores chemical depolymerization-based recycling, where dynamic or reversible polymer networks are cleaved and reassembled to recover monomeric, oligomeric, or even polymer precursors for closed-loop regeneration.

Beyond their immediate environmental benefits, recycling-oriented strategies in membrane technology also offer long-term advantages for resource conservation and waste management. They contribute to mitigating environmental pollution, reducing dependence on non-renewable feedstocks, and addressing the interconnected global challenges of resource depletion, waste accumulation, and water scarcity. This marks a decisive shift from the traditional “use-and-dispose” model toward a circular membrane economy. They minimize dependence on virgin resources, reduce the carbon footprint of manufacturing, and alleviate the environmental burden associated with waste disposal. The following subsections provide an overview of recent progress in each area and critically discuss the primary limitations that currently hinder their large-scale adoption.

### 2.1. Down- and Upcycling of Fouled EoL Membranes

Down- and upcycling of fouled membranes constitute one of the most practical and old strategies for extending the life cycle of polymeric membranes and reducing solid waste generation. Upcycling refers to the conversion of used or fouled low-pressure membranes, such as MF or UF membranes, into higher-performance NF or RO membranes through additional modification steps. This is typically achieved by forming a new selective layer on the used substrate via the interfacial polymerization (IP) technique [41,42] or other coating methods, allowing the recovered membrane to operate at higher selectivity and pressure levels than its original configuration.

Conversely, downcycling involves the conversion of high-pressure membranes, such as RO, into NF or lower-pressure membranes (UF or MF) once their selective layers are irreversibly fouled. This is often accomplished by chemical removal or treatment of the top layer, thereby repurposing the remaining membrane part for less demanding separations. Both approaches maximize material utilization and reduce the demand for virgin polymers and the carbon footprint associated with new membrane fabrication.

Several techniques have been developed for the downcycling of fouled RO membranes, each aiming to recover functional materials or convert deteriorated membranes into useful lower-pressure variants. Among these techniques, both direct oxidation and sequential alkaline pre-treatment followed by exposure to oxidizing agents are among the most extensively studied and effective strategies. In the latter two-step process, an alkaline pre-treatment (e.g., NaOH exposure) and a subsequent oxidative exposure work synergistically to remove fouling layers and selectively degrade the PA selective layer. The alkaline stage plays a crucial preparatory role as it loosens and partially dissolves inorganic and organic foulants, weakens the adhesion between the fouling layer and the membrane surface, and swells the PA structure to improve the accessibility of subsequent oxidants. Furthermore, under alkaline conditions, many organic foulants such as humic substances and biofilms become more soluble, while metal hydroxides and other inorganic precipitates are destabilized. Following this pre-treatment, the membrane becomes chemically conditioned for exposure to oxidative agents, enabling a controlled and uniform reaction with the selective layer. Both techniques exploit the chemical sensitivity of the PA selective layer to oxidative agents such as sodium hypochlorite (NaClO) conditions [43,44,45,46,47,48,49,50], potassium permanganate (KMnO_4_) [51,52,53], hydrogen peroxide (H_2_O_2_) [53], and Ozone (O_3_) [54], allowing the controlled removal or thinning of the active layer while preserving the integrity of the underlying membrane support. 

In a recent study, Wang et al. demonstrated the systematic downcycling of fouled RO membranes into functional UF and NF membranes following the two-step process [55]. In their study, a DOW Fortilife^TM^ XC70 RO module heavily fouled with Silicon and Aluminum complexes (Si-Al complexes) was used. The Si-Al complex foulants that covered the EoL RO membranes were partially dissolved and transformed in 0.10 M NaOH solution. Following this, the partially cleaned RO membrane was exposed to 0.50% NaClO solution for 0, 2, 4, and 6 h, leading to the degradation of the PA layer. Both alkaline pre-treatment and NaClO exposure time had a major influence on the membrane performance (i.e., UF or NF). 

For example, short NaClO exposure induces localized damage or defects in the PA layer, converting the alkaline-pre-treated RO structure into a loose NF-like membrane. Prolonged exposure results in the complete degradation of the PA layer, yielding a UF-like membrane. However, when NaClO is applied directly to fouled RO membranes without alkaline pre-treatment, the Al^3+^ present in the Si-Al complex accelerates uncontrolled PA oxidation, producing what Wang et al. described as an “undesired membrane” [55]. Furthermore, the effect of alkaline pre-treatment on the downcycling performance was systematically examined through comparative analysis of membranes treated with and without NaOH solution. When fouled RO membranes were directly exposed to NaClO oxidation without prior alkaline pre-treatment, a significant increase in water permeance was accompanied by a rapid decline in Na_2_SO_4_ rejection (e.g., <<40.0%) as the exposure time increased. This behavior indicated uncontrolled degradation of the PA layer caused by the presence of Si-Al complex foulants, which interfered with the oxidation reaction and led to uneven removal of the selective layer, producing membranes with UF-like characteristics but poor selectivity [55]. 

In contrast, the introduction of a 24 h alkaline pre-treatment before NaClO exposure yielded markedly improved control over the degradation process. Under these alkaline conditions, the solubility of silica increases, promoting the dissolution of Si-rich components, while Al-based compounds such as Al_2_O_3_ and Al_2_O_3_·SiO_2_ were converted into amorphous Al-O-Si oxides. This transformation facilitates foulant removal and prevents Al^3+^-catalyzed oxidative degradation of the PA layer in the following step. Subsequent exposure to NaClO for 2–4 h produced membranes with loose NF-like behavior (~70.0% Na_2_SO_4_ rejection), while prolonged exposure of about 6 h resulted in complete degradation of the PA layer and UF-like performance [55].

**Figure 2:**
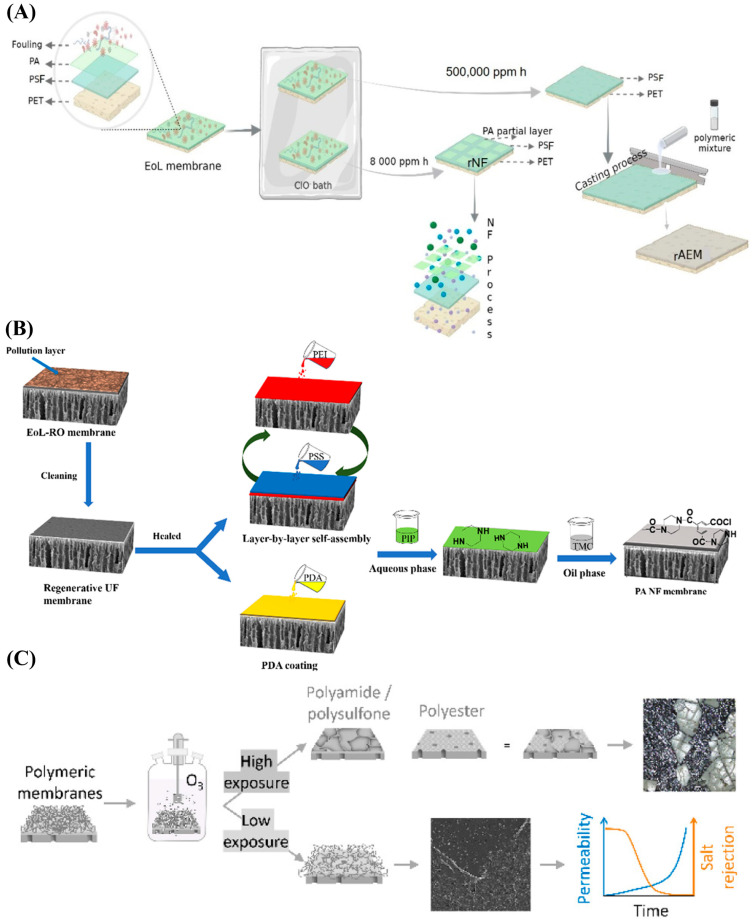
(**A**) EoL RO membrane downcycling to NF-like membrane and AEM, (**B**) EoL RO membrane downcycling to NF-like membrane with healing step, and (**C**) low and high membrane ozonation to produce downcycled membranes. The schematics in (**A**) were reproduced or adapted with permission from ref. [56], in (**B**) were (reprinted (“Adapted” or “in part”) with permission from Ref. [57], Copyright 2023 American Chemical Society., and in (**C**) were reprinted (“Adapted” or “in part”) with permission from Ref. [54], Copyright 2025 American Chemical Society.

In another work, Pompa-Pernía et al. validated the systematic transformation of EoL RO membranes into NF-like and anion-exchange membranes (AEM) through a direct NaClO oxidation process, as schematically shown in Figure 2A [56]. The membrane used in this process was sourced from a TM720-400 EoL seawater RO module supplied by Toray Industries, Inc. The NF-like membrane was prepared by partial oxidative thinning of the PA layer using a direct 8000 (mg/L)·hour NaClO exposure at room temperature, allowing the complete removal of foulants and the production of a membrane with NF-like properties. The membrane showed a steady state permeance of 4.89 LMH/bar with ion rejection of 96.0%, 80.0%, 66.4%, and 74.5% for SO_4_^−2^, Cl^−1^, NO_3_^−1^, and Na^+1^, respectively.

The same EoL RO membrane was utilized for full oxidative removal of the PA layer at 500,000 (mg/L)·hour NaClO exposure, as shown in Figure 2A, followed by deposition of a mixture of polyvinyl chloride (PVC) and Amberlite^®^ IRA-402 layer, converting the membrane into an anion-exchange membrane (AEM) suitable for ED applications [56]. When tested for the demineralization of synthetic urban wastewater, the AEMs derived from recycled RO modules achieved a 60.0% demineralization rate with an energy consumption below 1.50 kWh/m^3^, compared to 1.06 kWh/m^3^ for their commercial counterparts. 

These results indicate that some recycled membranes derived from EoL RO membranes exhibited slightly lower performance compared to their commercial counterparts. This could be due to the incomplete structural recovery and residual defects inherited from the original EoL membrane. During the oxidation process, the polyamide layer is fully removed, and while this exposes the porous substrate for subsequent modification, it can also leave behind microporous irregularities, weakened interfaces, and local chemical damage within the support structure. These imperfections compromise the uniformity of the newly deposited membrane layer, collectively lowering the overall efficiency. Such findings suggest that a healing step may be necessary before converting recycled RO membranes into functional membranes.

In this context, Cui et al. developed a method for oxidation-healing-IP strategy to regenerate EoL RO membranes into high-performance NF membranes [57]. As shown in Figure 2B, the TMH10A-400 EoL SWM RO module supplied by Toray Industries, Inc. was first cleaned using a mixture of 5.00 wt.% NaOH, 2.00 wt.% sodium cumene sulfonate, 5.00 wt.% ethylene diamine tetra acetic acid (EDTA), 5.00 wt.% sodium citrate dihydrate, and water. The membrane was then oxidatively treated with 12,385 mg/L NaClO solution for 60 h to remove the PA layer, yielding a porous UF substrate. Following this, a two-step surface repair strategy was implemented, as illustrated in Figure 2B. First, a polydopamine (PDA) coating was applied to the UF substrate, introducing abundant hydrophilic functional groups that enhanced surface wettability and acted as an adhesive interlayer. Subsequently, a layer-by-layer (LbL) self-assembly of polyethyleneimine (PEI) and poly(styrene sulfonate) (PSS) was performed to further reconstruct and functionalize the surface (Figure 2B). Each cation/anion deposition cycle represented one bilayer of polyelectrolytes, while an additional cationic deposition formed a half-bilayer (N+0.5) configuration. Thus, N-bilayer membranes contained N pairs of PEI/PSS coatings, whereas N+0.5 bilayer membranes featured a terminal PEI layer. Following this healing treatment, the membranes underwent conventional IP to fabricate the regenerated NF-like membrane [57].

This modular LbL healing approach was effective in restoring the mechanical and interfacial properties of the degraded substrate, leading to enhanced adhesion of the subsequent PA layer. Among the resulting membranes, the 2.0-bilayer system demonstrated a good balance of permeability and selectivity, achieving a permeance of 7.25 LMH/bar, which is slightly higher than the pristine RO membrane, and ion rejections of 99.0%, 99.0%, 56.5%, and 71.4% for Na_2_SO_4_, MgSO_4_, NaCl, and MgCl_2_, respectively. While this study effectively demonstrated the feasibility of regenerating high-performance NF-like membranes through a sequential PDA and LbL surface-healing process, it also presents certain limitations. Notably, no direct comparison was made with pristine or commercial NF membranes, which makes it difficult to assess the true competitiveness and practical viability of the recycled membranes. Without benchmarking against established commercial standards, it remains unclear whether the recovered membranes can match the selectivity, permeability, and long-term stability expected in commercial or pristine NF membranes.

In addition to chlorine-based oxidation, ozone (O_3_) has recently shown slight potential as a promising oxidant for downcycling EoL RO membranes, as illustrated in Figure 2C. Zappulla-Sabio et al. investigated O_3_ as a non-halogenated oxidizing agent capable of degrading the PA active layer in both RO and NF membranes [54]. Their study evaluated both new membranes (SW30HRLE, BW30, NF90, and NF270) and used EoL modules (SW30HRLE-400-ilec and BW30-400-FR), allowing direct comparison between pristine and aged materials under identical O_3_ treatment conditions.

Initial assessments with O_3_-enriched water (20.0 mg/L O_3_) exposure for varying durations (0.5 h, 1 h, and 3 h) revealed that the oxidizing strength of O_3_ led to pronounced degradation of the PA layer, especially at longer contact times [54]. SEM characterization showed that, even after 30 min, the PA layer began to lose its continuity, displaying early signs of cracking. After 1 h of treatment, substantial deterioration of the selective layer was evident, with widespread cracking and pore enlargement indicative of over-oxidation. Notably, after 3 h of O_3_ exposure, complete delamination of the active layer occurred in membranes retrieved from used BW30 RO modules, where the PET support layer became clearly visible under SEM [54]. This observation signifies the full removal of the PA selective layer and the degradation of the intermediate PSf layer, suggesting that excessive O_3_ exposure can penetrate through the entire composite structure. The PET layer’s exposure thus serves as direct evidence of uncontrolled oxidation and loss of the membrane’s functional integrity under prolonged O_3_ treatment.

Under low ozone treatment conditions, specifically exposure to a 3 mg/L O_3_-enriched water solution for 1, 5, 15, and 30 min, the degradation of the PA layer was more controlled compared to high-dose treatments [54]. At shorter exposure times (1–5 min), the PA surface showed only minor oxidative etching and roughening. As the exposure time increased to 15–30 min, a more pronounced degradation of the PA layer was observed. Most importantly, all membranes exhibited a significant reduction in NaCl rejection, even after just 1 min of exposure to the 3 mg/L O_3_ solution. With continued exposure, the effect became more pronounced as many membranes lost nearly all NaCl rejection capability after only 5 min of treatment [54]. This rapid decline indicates that O_3_, even at low concentrations, can aggressively attack the amide linkages in the PA network, compromising the membrane’s selective barrier. 

Overall, these results emphasize that, although low-dose O_3_ treatment offers a more controlled structural response than high-dose oxidation, it still requires careful optimization of concentration, exposure time, and operating conditions to balance permeability enhancement with the preservation of salt selectivity. Additional studies from the literature on downcycling EoL RO membranes into functional membranes are summarized in Table 1.

**Figure 3:**
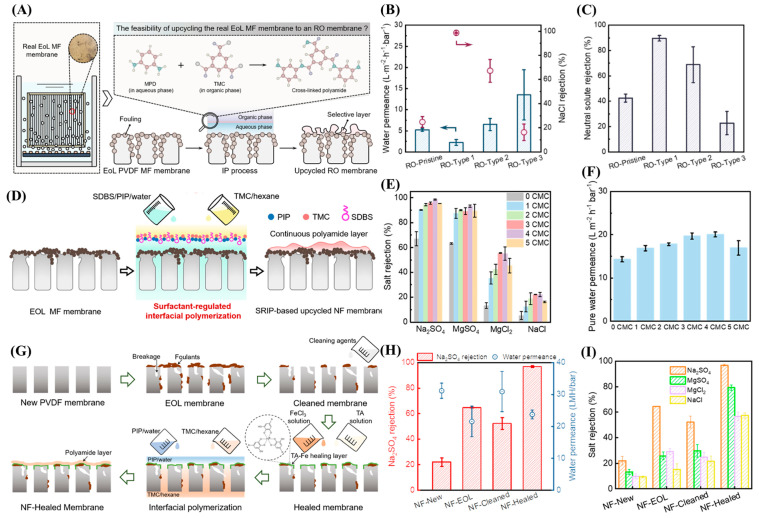
(**A**) Schematic of the EoL MF membrane upcycling to RO-like membrane, RO membranes performance in terms of (**B**) water permeance and NaCl rejection, and (**C**) neutral solute rejection. (**D**) Schematic of the EoL MF membrane upcycling to NF-like membrane using surfactant-regulated IP technique, NF membranes performance in terms of (**E**) salts rejection, and (**F**) water permeance. (**G**) Schematic of the EoL MF membrane upcycling to NF-like membrane using intermediate healing step, and NF membranes performance in terms of (**H**) water permeance and Na_2_SO_4_ rejection, and (**I**) salts rejections. Figures (**A**–**C**) are reprinted (“Adapted” or “in part”) with permission from Ref. [59]. Copyright 2025 American Chemical Society, Figures (**D**–**F**) are reprinted (“Adapted” or “in part”) with permission from Ref. [28]. Copyright 2023 American Chemical Society. Figures (**G**–**I**) are reprinted (“Adapted” or “in part”) with permission from Ref. [60]. Copyright 2022 American Chemical Society.

Similarly to membrane downcycling, EoL UF or MF membranes can be upcycled into NF- or even RO-like membranes through various surface modification techniques. One such approach involves the direct creation of a PA layer onto the EoL UF or MF substrate. For instance, Chen et al. employed EoL PVDF MF membranes retrieved from wastewater treatment plants at different operational positions, terminal (Type 1), middle (Type 2), and front (Type 3), to fabricate RO-like membranes via an IP process, as illustrated in Figure 3A [59]. Prior to IP, the membranes underwent a rigorous cleaning sequence involving 2 h of NaClO treatment followed by 1.50 wt.% citric acid washing for an additional 2 h. Despite this treatment, a fraction of the foulants remained, and the IP was therefore performed on partially fouled membranes using 2.0 wt.% m-phenylenediamine (MPD) in water and 0.15 wt.% TMC in hexane [59]. The resulting membranes were denoted as RO-Type 1, RO-Type 2, RO-Type 3, and RO-Pristine (the latter referring to IP conducted on a pristine PVDF substrate).

Among these, the RO-Type 1 membrane demonstrated the highest NaCl rejection (98.6%) with a water permeance of 2.30 LMH/bar, as shown in Figure 3B. In contrast, RO-Type 2 and RO-Type 3 exhibited higher permeance but significantly lower salt rejection, highlighting the influence of the underlying fouling characteristics on PA layer formation. Notably, the Type 1 membrane, collected from the terminal section of the treatment system, exhibited greater irreversible fouling despite the intense chemical cleaning, which provided a denser and more favorable surface for uniform PA layer growth during IP. This relationship between fouling state and selective layer morphology was further supported by neutral solute rejection experiments, which showed enhanced molecular sieving in RO-Type 1 membranes (Figure 3C). Overall, the findings emphasize that residual fouling within EoL membranes can positively influence PA layer formation, enabling the fabrication of high-performance RO-like membranes directly from used MF substrates.

Building upon this concept, researchers have further explored methods to intentionally regulate the surface properties of EoL membranes to achieve more uniform and defect-free PA layers. In this context, Dai et al. developed a surfactant-regulated interfacial polymerization (SRIP) approach as a one-step upcycling strategy for converting fouled EoL PVDF MF membranes into NF-like membranes, as shown in Figure 3D [28].The study identified that the surface hydrophobicity of EoL membranes, induced by both the polymer matrix and residual foulants, hinders uniform aqueous monomer uptake and thus limits PA layer formation. To overcome this, they incorporated sodium dodecylbenzene sulfonate (SDBS), a commercially available anionic surfactant, into the aqueous piperazine (PIP) solution to enhance substrate wettability, increase monomer adsorption, and promote the growth of a continuous PA layer without the need for prior chemical cleaning or modification [28].

The as-fouled EoL MF membranes were only rinsed with clean water. Following this, an IP process was performed using 0.20 wt./v% PIP aqueous solution with a 0.16 wt./v% TMC/Hexane to form a thin PA selective layer over the fouling layer [28]. In the SRIP process, SDBS was added to the PIP solution at concentrations ranging from 0 to 5 times its critical micelle concentration (CMC), producing a series of upcycled NF membranes with tailored surface morphologies and separation properties. Their results revealed that the 4 times CMC formulation achieved optimal performance, yielding an upcycled NF-like membrane with a water permeance of 20.1 LMH/bar and Na_2_SO_4_ rejection of 98.6% (Figure 3E,F), significantly higher than that obtained via conventional IP on fouled substrates that had Na_2_SO_4_ rejection of 67.1%. Higher surfactant concentrations (e.g., 5 CMC) caused excessive micelle formation, which disrupted polymerization and introduced surface defects. Mechanistic insights confirmed that SDBS reduced the interfacial tension (from ~78.4 to 70.9 mN/m) and enhanced PIP monomer enrichment near the interface, facilitating the formation of a denser and more crosslinked PA network. Collectively, this surfactant-assisted strategy demonstrates a more straightforward method for upcycling fouled EoL MF/UF membranes.

In some cases, an intermediate healing step of the membrane substrate may be necessary to enhance surface integrity, reduce defect density, and ensure the uniform formation of a new selective layer. Wang et al. employed this on a cleaned EoL MF membrane using a tannic acid–iron (TA-Fe) complex treatment shown in Figure 3G [60]. The fouled MF membrane was first chemically cleaned using an oxidative-acidic sequence, typically NaOCl followed by oxalic acid, to remove organic and inorganic foulants. The cleaned membrane was then immersed sequentially in 0.19 wt.% FeCl_3_ for 30 s and 0.20 wt.% TA for 1 min. During this step, a TA-Fe coordination complex forms on the membrane surface, bridging damaged or enlarged pores and creating a uniform, hydrophilic coating [60]. This metal–organic complex acts as a healing layer, effectively repairing microdefects and narrowing surface pores, which would otherwise hinder the formation of a defect-free PA layer. After the intermediate healing step, the membrane undergoes IP between 0.40 wt./vol% PIP in water and 0.32 wt./vol% TMC in hexane, producing a thin, dense PA selective layer atop the healed substrate.

As a reference, the same IP procedure was also carried out on new, unmodified EoL, and chemically cleaned EoL membranes, which were designated as NF-New, NF-EoL, and NF-Cleaned, respectively, to assess the contribution of the healing step (NF-Healed). The healed NF membrane exhibited a water permeance of ~23.7 LMH/bar and Na_2_SO_4_ rejection of around 96.9% (Figure 3H), significantly outperforming the rejection capabilities of NF-EoL (64.6%), NF-New (22.1%), and NF-Cleaned (32.2%) [60]. The same membrane also showed higher NaCl, MgCl_2_, and MgSO_4_ rejections than the other NF membranes, as shown in Figure 3I. The enhanced rejection and permeability were attributed to the synergistic effects of surface healing and controlled fouling-layer chemistry, where the TA-Fe complex and residual hydrophilic foulants on the EoL MF substrate facilitated greater PIP monomer adsorption prior to IP [60]. This led to the formation of a more uniform and densely cross-linked PA layer, resulting in better ion rejection and water permeance compared to the membranes fabricated on new or simply cleaned substrates. Overall, this cleaning-healing-upcycling sequence highlights the importance of surface restoration in achieving high-quality upcycled membranes.

Another widely applied approach in the upcycling of EoL MF and UF membranes is the LbL self-assembly of polyelectrolytes, which was previously discussed in the context of converting downcycled EoL RO membranes into NF membranes. In that case, EoL RO membranes were exposed to high doses of oxidative agents for extended durations, leading to the complete removal of the PA selective layer and leaving behind a porous UF or MF substrate suitable for subsequent LbL modification. In the upcycling approach, the process begins with an EoL UF or MF membrane that is chemically cleaned, regenerated, and prepared for the deposition of polyelectrolyte multilayers to build a new selective layer. 

For example, Malaisamy and Bruening developed polyelectrolyte multilayer NF membranes by depositing alternating layers of oppositely charged polymers on PES UF substrates with different molecular weight cutoffs (MWCOs) [61]. In their work, membranes were fabricated by sequentially assembling 1.50–4.50 bilayers of PSS with either protonated poly(allylamine) (PAH) or poly(diallyldimethylammonium chloride) (PDADMAC), using 0.02 M polymer solutions and deposition times of 2–5 min per layer. Each membrane was capped with a terminal PSS layer to ensure a negatively charged surface and improved fouling resistance.

Performance evaluation revealed that on a 50.0 kDa UF PES substrate, a membrane containing 3.50 bilayers of PSS/PAH achieved a water permeance of approximately 12.4 LMH/bar, with SO_4_^−2^ and sucrose rejections of 93.4% and 96.0%, respectively [61]. Similarly, the 3.50-bilayer PSS/PDADMAC configuration demonstrated a slightly higher permeance of around 15.6 LMH/bar while maintaining comparable SO_4_^−2^ (95.0%) and sucrose (92.0%) rejections. These results underscore the ability to fine-tune membrane selectivity and permeability through precise control over the number and composition of polyelectrolyte bilayers, highlighting the versatility of such an assembly technique for fabricating NF-like membranes from EoL UF membranes. Other studies have also reported the successful upcycling of membranes aimed at advancing circularity in membrane technology [26,62,63].

Despite these promising outcomes, the practical deployment of upcycling and downcycling strategies remains constrained by several critical limitations. On the positive side, these approaches offer clear environmental and economic advantages, they extend the service life of polymeric membranes, reduce plastic waste generation, and lower the need for virgin polymer synthesis. Upcycling, in particular, adds further value by converting low-pressure membranes into high-selectivity systems, while downcycling provides a viable use for structurally degraded RO or NF membranes in less demanding operations such as pre-treatment or wastewater polishing. Collectively, such routes advance the broader vision of a circular membrane economy, emphasizing reuse rather than replacement. However, practical and scalability challenges hinder the transition of these laboratory-scale demonstrations to real industrial adoption. Most current studies rely on disassembling EoL SWM modules, manually cutting open housings, and extracting individual membrane sheets for chemical or physical treatment. While effective for controlled experimentation, this process might become labor-intensive, time-consuming, and destructive, preventing reintegration of the recycled membrane into the original module. 

Moreover, the chemical cleaning and re-functionalization stages commonly employed during upcycling and downcycling, typically involving strong oxidants (e.g., NaOCl), acids, bases, and surfactants, pose additional risks to the membrane’s physical integrity. These harsh treatments, while effective at removing foulants or preparing the surface for modification, can degrade the polymer backbone, alter pore morphology, or weaken the interfacial adhesion between the active and support layers. As a result, the mechanical strength, flexibility, and compaction resistance of the recycled membranes may be significantly reduced, even if their separation performance appears improved in the short term.

A major concern in the current body of literature is that mechanical robustness is rarely evaluated following these regeneration or upcycling procedures. Most studies focus almost exclusively on water permeance, solute rejection, and antifouling behavior, while neglecting to assess tensile strength, elasticity, burst pressure, or structural resistance, parameters that are critical for reliable operation under real feed pressures. This omission undermines the practical reliability of many reported recycling methods. A membrane that performs well in bench-scale filtration tests but lacks sufficient mechanical stability cannot withstand prolonged use in pressurized systems or full-scale modules.

Furthermore, the non-uniform fouling patterns within full-scale modules further complicate treatment consistency, as performance recovery tends to vary across membrane areas exposed to different feed conditions. The concept of direct recycling within intact SWM modules, without dismantling, offers an appealing solution, but it has received limited attention. Only a few studies, such as that by García-Pacheco et al. [48], have successfully demonstrated whole-module recycling using a large reactor tank system. Such approaches are promising for minimizing manual intervention and preserving module structure, yet systematic optimization and scalability studies remain lacking. Therefore, more focus on developing module-compatible upcycling protocols, optimizing reagent flow and reaction kinetics within SWM architectures, and integrating continuous regeneration systems to enable practical, cost-effective, and environmentally responsible implementation at a larger scale.

### 2.2. Recycling of Plastic Waste for Membrane Fabrication

**Figure 4:**
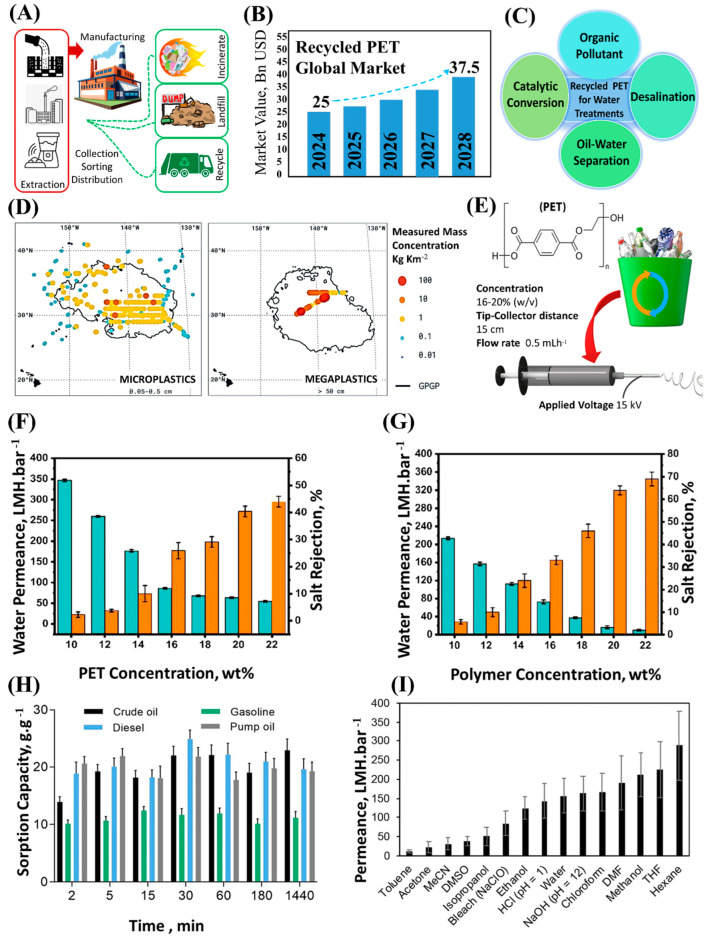
(**A**) Schematic illustration showing the emergence of plastic waste. (**B**) Global recycled plastic market (raw data from Ref. [67]). (**C**) Applications of plastic waste for water treatments. (**D**) Comparison of modeled and measured mass concentrations in the Great Pacific Garbage Patch, showing various plastic waste forms by size. (**E**) Illustration of the fabrication process of rPET membranes. (**F**) Effect of rPET concertation on water permeance of and NaCl rejection of membrane. (**G**) Effect of rPET concentration when rPET membranes were used as a support for IP process. (**H**) Sorption performance of the rPET-based membrane for different oils over time. (**I**) Permeance of 10 wt.% rPET membrane measured with different solvents. Figures in (**A**) are reproduced or adapted with permission from Ref. [64], Copyright 2024 Springer Nature. Figures shown in (**D**) are reproduced or adapted with permission from Ref. [70], Copyright 2018 Springer Nature. Figures shown in (**E**,**H**) are reprinted (“Adapted” or “in part”) with permission from Ref. [74]. Copyright 2022 American Chemical Society. Figures shown in (**F**,**G**) are reprinted (“Adapted” or “in part”) with permission from Ref. [76]. Copyright 2023 American Chemical Society. Figures shown in (**I**) are reprinted (“Adapted” or “in part”) with permission from Ref. [77]. Copyright 2019American Chemical Society.

Global plastic production has exceeded 360 million tons annually, yet only a small fraction, ~9.00%, is recycled into circular material streams, while the majority is either landfilled or released into the environment (Figure 4A) [64]. Although mechanical recycling remains the predominant route, it faces persistent technical and economic challenges, including contamination, polymer mixing, and degradation of physical properties that compromise product quality and elevate processing costs [65]. In contrast, advanced recycling techniques such as chemical depolymerization, solvent purification, and catalytic or thermochemical conversion enable higher-value recovery but remain constrained by scalability, operational complexity, and high energy demand [66]. 

As shown in Figure 4B, the global market for recycled plastics continues to expand, projected to grow at 8.10% annually and reach USD 114.8 billion by 2033, with recycled polyethylene terephthalate (rPET) representing the largest share [67]. rPET, a chemically stable thermoplastic polyester, has gained considerable attention for its potential in membrane-based separation processes, including desalination, oil–water separation, catalytic conversion, and organic pollutant removal (Figure 4C). The predominant source of rPET feedstock is single-use beverage bottles, which constitute one of the most abundant plastic waste streams globally [68]. Studies further report that microplastics account for the largest fraction of rPET waste, while sand-based filtration systems have proven effective in their recovery, supporting the case for rPET as a promising, regionally abundant feedstock for membrane fabrication (Figure 4D).

Plastic recycling encompasses a broad spectrum of technological pathways, from mechanical reprocessing and thermochemical conversion to solvent- or chemically driven depolymerization and emerging biocatalytic degradation approaches. Each route presents trade-offs in energy intensity, process cost, and product quality. Aligning these techniques with circular economy principles requires a shift toward material designs that prioritize recyclability, reusability, and resource recovery [64]. Among these approaches, plastic pyrolysis remains particularly debated due to its high energy consumption, emission concerns, and limited economic feasibility, with critics arguing that its focus on fuel recovery diverges from true carbon-neutral goals [69]. Comprehensive and transparent life cycle assessments (LCAs) are therefore needed to accurately quantify the environmental and economic impacts of each recycling route within a circular plastics framework.

Recent studies demonstrate that recycling plastic waste into functional membranes offers a technologically viable and sustainable pathway for circular materials integration [70]. Spent membrane modules have also been repurposed into reusable polymer feedstocks [71], which can be reprocessed into functional membranes or catalytic supports. However, residual contaminants, such as fluorescent dyes and additives, often require removal prior to use in water treatment or separation applications [72]. Among available plastic wastes, rPET is particularly attractive, as it can be converted into efficient filtration membranes via electrospinning or phase inversion (Figure 4E) [72,73,74]. This approach reduces dependence on virgin polymers, lowers carbon footprint, and produces high-value materials suitable for diverse water purification systems.

Most reports employ rPET concentrations of 10.0–15.0 wt.% dissolved in trifluoroacetic acid/dichloromethane (TFA/DCM) mixtures [72,75,76]. For example, Hussain et al. recycled rPET bottles into asymmetric membranes for desalination via the NIPS method, systematically optimizing polymer content, co-solvent ratio, additive concentration, and coagulation bath temperature to tailor performance [76]. They also upcycled the rPET membranes into NF-like membranes via IP of 2.00 wt.% PIP and 0.20 wt.% TMC on the porous rPET membrane. Their results showed that increasing rPET concentration from 10.0 to 22.0 wt.% reduced water permeance from 346.9 to 55.1 LMH/bar while NaCl rejection rose from 2.25 to 44.0%, when keeping the coagulation bath temperature at 25 °C (Figure 4F) [76]. Higher polymer concentrations led to denser structures because of the higher viscosity of the casting solution. When IP was carried out on these membranes, the permeance decreased further, but NaCl rejection increased noticeably, as shown in Figure 4G. For example, an NF-like membrane created on a 22.0 wt.% rPET membrane exhibited a water permeance of 10.2 LMH/bar with NaCl rejection of 69.0% [76]. Overall, the enhanced rejection and ion selectivity are influenced by Donnan exclusion, where hydrolyzed acyl chloride groups form negatively charged carboxyl sites that repel chloride ions and restrict sodium transport to maintain electroneutrality. This combined steric and electrostatic control underpins the observed improvement in NaCl rejection, underscoring the potential of recycled PET-based membranes for efficient, durable separation processes [76].

Beyond desalination, rPET-based membranes exhibit strong hydrophobicity (water contact angle ~137°) and high oleophilicity, enabling efficient oil adsorption. Topuz et al. fabricated nanofibrous adsorptive membranes from recycled PET bottle wastes via electrospinning using TFA and TFA/DCM (70/30 *v*/*v*%) solvent systems (Figure 4E) [74]. By tuning PET concentrations (80–200 g/L), they achieved bead-free, uniform nanofibers with diameters between 140 and 490 nm and excellent mechanical flexibility. As shown in Figure 4H, the rPET membrane exhibited sorption capacities of 14.0 ± 0.90 g/g (crude oil), 18.8 ± 2.00 g/g (diesel), 20.6 ± 1.20 g/g (pump oil), and 10.1 ± 0.60 g/g (gasoline) within 2 min. 

This quick sorption is directly related to their strong oleophilicity, which allowed rapid spreading of oil droplets on the membrane surface within seconds. Furthermore, this strong oil uptake originates from van der Waals and hydrophobic interactions between nonpolar hydrocarbons and aromatic PET rings, enabling both surface adsorption and diffusion within the fibrous matrix [74]. The higher affinity toward diesel reflects stronger interactions with higher molecular weight hydrocarbons, while crude oil reached similar saturation after 24 h due to its slower diffusion [74]. Lastly, the membranes were mechanically reusable, retaining over 75.0% of their initial sorption capacity after five cycles of oil recovery, demonstrating that rPET nanofibers can act as lightweight, flexible, and sustainable sorbents for mitigating oil spills.

The rPET membranes also exhibited outstanding chemical resilience. For example, Pulido et al. fabricated solvent-resistant membranes from recycled PET bottles via the NIPS method and evaluated them for UF performance under harsh conditions [77]. The membranes were prepared using 10.0–14 wt.% PET dissolved in TFA/DCM containing 2.00–6.00 wt.% PEG as a pore-forming additive and immersed in either ethanol or water with and without evaporation time. Performance of the membrane obtained with 10.0 wt.% PET dissolved in TFA/DCM containing 4 wt.% PEG, immersed in ethanol, and without any evaporation time (denoted as M8 in their study) is shown in Figure 4I. This membrane displayed a pore size distribution mostly between 75.0–85.0 nm, ethanol permeance of 98.0 LMH/bar, and a molecular weight cutoff (MWCO) of 50 kDa in DMF, suitable for UF in nonaqueous media.

As shown in Figure 4I, the PET membranes exhibited variable permeance across solvents, highlighting solvent-dependent transport behavior linked to polymer-solvent affinity [77]. Furthermore, the membranes maintained structural integrity and recovered up to 90.0% of their original ethanol permeance after exposure to most solvents, confirming reversible compaction. They also resisted degradation in acidic and oxidative environments, reflecting their robust chemical and thermal stability [77]. 

These results highlight the robustness and versatility of rPET-based membranes, demonstrating their promising permeability, selectivity, and reusability across diverse water filtration applications. The growing body of research underscores the potential of plastic waste as a sustainable, recyclable, and abundant resource that can be upcycled into high-performance membranes. 

Despite these advances, recycling plastic waste for membrane fabrication faces substantial environmental and practical challenges that must be addressed before large-scale implementation becomes feasible. Environmentally, the widespread reliance on toxic and high-global-warming-potential solvents such as TFA and DCM undermines the sustainability of these processes, as their volatility, toxicity, and disposal difficulties offset some of the environmental gains achieved by recycling. Moreover, the energy-intensive nature of polymer dissolution, electrospinning, and solvent evaporation introduces significant carbon and operational footprints. From a practical perspective, achieving consistent feedstock purity and composition remains a major bottleneck; contamination from dyes, additives, and mixed polymer streams leads to variations in membrane morphology and mechanical strength. Without addressing these issues, particularly through green solvent substitution, solvent recovery integration, and standardized feedstock purification, the environmental advantages of rPET-based membrane fabrication risk becoming largely theoretical rather than practically sustainable.

### 2.3. Emerging Techniques in Membrane Recycling

While previous recycling strategies for used membranes have primarily centered on the upcycling or downcycling of membrane materials and the reuse of plastic waste, a new and rapidly emerging field is redefining how membrane materials can be recovered and reused. This new generation of techniques enables the complete disassembly of used membranes into their polymeric precursors, followed by refabrication into new membranes with comparable functionality and integrity. Among these emerging strategies, the incorporation of covalent adaptable networks (CANs), or more broadly, dynamic covalent polymers (DCPs), has gained particular attention. Although the chemistry underpinning DCPs and CANs is not new, their application in membrane fabrication and recycling is only beginning to emerge, marking a significant conceptual shift in how membrane materials are designed, used, and regenerated.

CANs and DCPs uniquely combine the structural robustness of thermosets with the reprocessability of thermoplastics, owing to the presence of reversible covalent bonds that can cleave and reform under external stimuli such as heat, pH, or chemical triggers [78,79,80]. These adaptable linkages endow the polymer network with constitutional dynamics, allowing the material to undergo controlled rearrangement without compromising its integrity [81]. Such features introduce a new paradigm in membrane design, one in which bond reversibility, rather than polymer blending or surface coating, dictates recyclability. The ability of these materials to undergo dynamic bond-exchange reactions under mild conditions allows fouled or aged membranes to be depolymerized and subsequently reconstructed into new membranes. This represents a fundamental transformation from the traditional linear life cycle of membranes to a truly circular one, where the same polymeric building blocks can be repeatedly reconfigured with minimal loss of function or mechanical strength.

**Figure 5:**
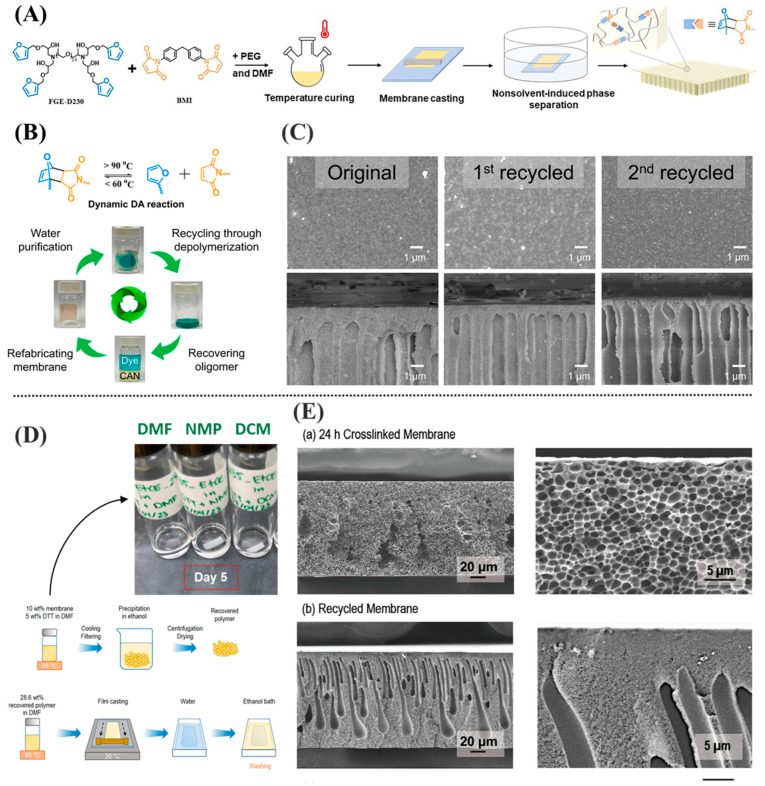
(**A**) Schematic illustration of the fabrication process of recyclable membranes, (**B**) illustration of the membrane recycling steps, (**C**) SEM images showing the top and cross-sectional views of the original and recycled membranes, (**D**) schematic of the membrane recycling with visual observation of the dissolution behavior in different solvents, and (**E**) cross-sectional SEM images comparing the morphology of the original and recycled membranes. Images shown in (**A**–**C**) are reproduced or adapted with permission from ref. [82]. The results shown in (**D**,**E**) are reprinted (“Adapted” or “in part”) with permission from ref. [83]. Copyright 2024 American Chemical Society.

Building on this concept, Li et al. developed a closed-loop recyclable membrane based on a temperature-reversible CANs with furan–maleimide Diels–Alder (DA) adducts, demonstrating that full membrane depolymerization and refabrication can be achieved while retaining comparable morphology and function [82]. As shown in Figure 5A, the recyclable membrane was prepared by reacting a four-arm furan-functionalized oligomer (FGE-D230) with 1,1′-(Methylenedi-4,1-phenylene) bismaleimide (BMI) while utilizing DMF and PEG. The asymmetric membrane was then prepared via the nonsolvent-induced phase separation (NIPS) membrane fabrication method. The resulting membrane exhibited excellent mechanical strength, hydrophilicity, and filtration performance [82]. For instance, the pure water permeability of the membrane was around 28.5 LMH/bar with 98.4% Rose Bengal (RB) dye rejection [82].

Crucially, the use of CANs endowed the membrane with thermal depolymerization capability, allowing it to be dismantled and reassembled without loss of functionality. As shown in Figure 5B, the recycling process involves heating the used membrane to approximately 140 °C in DMF solution to trigger the retro-DA reaction, thereby depolymerizing the membrane and releasing fouling deposits and contaminants. During this process, the membrane transitions from a solid state into a homogeneous liquid mixture containing both the dissolved oligomers and foulants (Figure 5B). This mixture is then filtered to remove large impurities and liquid-extracted to recover the purified oligomeric precursors, which can be reused to refabricate new membranes through the same DA crosslinking process. Figure 5C confirms through Scanning Electron Microscope (SEM) imaging that the recycled membranes retained comparable morphology and cross-sectional structure to the original, highlighting the reproducibility of the process.

A more recent study by Ramírez-Martínez et al. proposed a recyclable solvent-resistant membrane based on reversible and dynamic disulfide bonds crosslinking, enabling complete depolymerization and regeneration of poly (ether imide) (PEI; branded as Ultem) membranes while maintaining structural and functional integrity [83]. As shown in Figure 5D, the recycling process begins with immersing the used, cysteamine-crosslinked membrane in a reducing solution containing 5.00 wt./v% 1,4-dithiothreitol (DTT) in DMF at 60 °C. The authors initially evaluated NMP, DMF, and DCM as potential solvents for the reduction process and found that DMF provided the most effective dissolution of the crosslinked membrane, while NMP and DCM failed to achieve full solubilization even after extended immersion (Figure 5D). Through reductive cleavage of the disulfide bonds, the membrane gradually dissolves into a homogeneous polymer solution after 5 days, visually confirming the breakdown of the crosslinked network (Figure 5D). This reduction step effectively releases embedded foulants and enables recovery of the polymer matrix. The resulting solution is subsequently cooled, filtered to remove insoluble residues, and precipitated in ethanol, followed by repeated centrifugation and washing cycles to purify the recovered polymer. The solid polymer is then dried and reused to prepare a new dope solution in DMF for membrane refabrication via the NIPS method.

Interestingly, the cross-sectional SEM images (Figure 5E) revealed that the recycled membranes displayed a different morphology and pore structure compared to the original membranes. The authors attributed this to the partial functionalized of the recycled polymer by cysteamine, leading to a transition from the original sponge-like structure to a finger-like morphology [83]. Despite these structural variations, the recycled membranes maintained excellent solvent stability and filtration performance comparable to the original crosslinked PEI membranes. Performance evaluations revealed that the recycled membranes exhibited a slightly lower rejection of Direct Red 80 (DR80) dye but demonstrated a markedly higher permeance compared to the crosslinked PEI membranes (6.20 LMH/bar vs. 1.12 LMH/bar), a behavior primarily attributed to the increased pore interconnectivity and more open membrane structure formed during recasting [83]. 

A more recent study showed that NF membranes can be fabricated from a novel polyester and PA pair derived from a recyclable monomer, which could be depolymerized back into the same monomer [84]. Although the authors did not reuse the recovered monomer to fabricate new NF membranes, they noted other potential applications. Overall, this closed-loop regeneration strategy leverages the dynamic reversibility of bonds, offering an effective route toward sustainable membrane materials that combine solvent resistance, recyclability, and reprocessability without significant loss of mechanical integrity or filtration efficiency. 

More studies have explored similar systems employing diverse chemistries and membrane configurations, from flat-sheet to electrospun architectures, to demonstrate the broader applicability of reversible covalent networks in membrane recycling and reprocessing. A summary of representative studies applying this concept in membrane fabrication is presented in Table 2, outlining the polymers used, recycling mechanisms, membrane types, and key performance outcomes.

Although this field is still in its early stages, it is natural that several challenges persist before such recyclable membrane systems can evolve from laboratory demonstrations to industrial reality. For example, most dynamic polymer-based recycling processes rely on temperature depolymerization (e.g., 140 °C or 120 °C) to trigger reversible bond cleavage and network reconfiguration, which increases energy consumption and raises concerns regarding polymer stability after repeated cycles. In addition, the use of hazardous organic solvents such as DMF, NMP, or DCM remains prevalent, undermining the environmental benefits of recyclability and complicating waste management. Replacing these with non-toxic solvents is therefore a cornerstone of green innovation, offering pathways to reduce occupational risks, enhance recyclability, and cut overall energy use.

Furthermore, the multi-step purification procedures required to separate foulants and recover reusable polymer/oligomers—typically involving sequential filtration, solvent extraction, and evaporation—add process complexity, solvent dependency, and operational cost. Although effective at the lab scale, these steps pose serious challenges to automation and large-scale implementation. Repeated cycles of heating, solvent exposure, and extraction may also alter polymer chain integrity, viscosity, or composition, thereby affecting the consistency of refabricated membranes over time. Nevertheless, these limitations should be regarded as engineering barriers rather than conceptual setbacks. The development of recyclable membranes based on dynamic covalent chemistries has already demonstrated that circularity can be built into the molecular design of separation materials. Continued innovation in low-energy bond-exchange systems, green solvent formulations, and integrated recovery processes will be essential to bridge the gap between lab feasibility and industrial sustainability. Together, these advances signal a decisive shift in the membrane field, from one centered on performance alone to one equally defined by recyclability, reusability, and resource efficiency.

## 3. Conclusions and Future Recommendations

The concept of recyclability in membrane technology has evolved from a niche research focus into a cornerstone of sustainable membrane manufacturing. The examined studies in this review clearly demonstrate that EoL membranes can be re-engineered into functional membranes through upcycling, downcycling, or full chemical recycling routes. Upcycling, particularly through IP or surface reconstruction, enables the transformation of low-pressure MF/UF membranes into high-performance NF- or RO-like membranes [59,60], while downcycling offers a practical means of repurposing degraded RO/NF membranes for less demanding applications [55,56]. 

In addition, the recycling of plastic waste into nanofibrous electrospun membranes has emerged as a promising direction, enabling the conversion of discarded polymers, such as PET bottles, into high-surface-area, porous structures suitable for water treatment applications. These membranes with excellent properties provide an avenue for valorizing post-consumer plastic waste, making them viable alternatives to conventional polymeric membranes.

In parallel, the recent emergence of CANs or, more generally, DCPs-based membranes introduces a molecularly circular design paradigm, allowing complete depolymerization and reformation within closed-loop systems [83,85]. Collectively, these approaches signify substantial progress toward a sustainable circular membrane economy that emphasizes material recovery, resource efficiency, and waste minimization. 

However, the industrial implementation of these recycling strategies remains limited due to several technical and practical challenges. Downcycling, despite being validated at laboratory and to some extent, pilot scales, lacks long-term operational data and standardized protocols for full-scale applications. Key knowledge gaps include an incomplete understanding of foulant–polymer interactions, insufficient cost–benefit and life cycle assessments (LCAs), and the absence of techno-economic frameworks quantifying the true environmental gains of recycled modules. Furthermore, most reported studies rely on manual disassembly of the SWM modules to extract the flat-sheet membranes for treatment. This practice, though effective experimentally, is labor-intensive, destructive, and impractical for industrial adaptation. More scalable routes, such as in situ regeneration of intact SWM modules, as demonstrated in the work of García-Pacheco et al. [48], should be prioritized for future research. 

Recycling PET still faces some key environmental and practical challenges, including the extensive use of toxic solvents (e.g., TFA, DCM), high energy consumption during polymer dissolution and processing, and difficulties in achieving consistent feedstock purity. From a broader sustainability perspective, the process offers considerable promise but introduces its own set of challenges, including unclear carbon footprint trade-offs.

Furthermore, emerging recycling techniques based on DCPs and CANs still face significant process-related limitations that impede large-scale adoption. These systems currently rely on multi-step recovery procedures involving depolymerization in toxic solvent at high temperatures, filtration, solvent extraction, and evaporation to isolate and purify the reusable oligomers/monomer or polymer. While these methods effectively demonstrate molecular circularity at the laboratory scale, they remain energy- and solvent-intensive, leading to high operational costs and low process efficiency. Moreover, repeated cycles of heating and solvent exposure can gradually affect polymer chain integrity and viscosity, compromising the reproducibility and stability of the refabricated membranes. 

Another major limitation lies in the neglect of mechanical and structural stability in current recycling studies. While most investigations emphasize water permeance, solute rejection, and antifouling behavior, few address tensile strength, compaction resistance, or fatigue durability after chemical cleaning or re-functionalization. The use of harsh oxidants, acids, or surfactants may compromise the polymer backbone and delaminate support layers, undermining long-term reliability. Without systematic mechanical testing, the practical deployment of recycled membranes in pressurized operations remains uncertain. Integrating comprehensive mechanical characterization and standardized durability metrics is, therefore, essential to validate the real-world feasibility of these processes.

Looking forward, several key research directions and technological priorities merit focused attention to accelerating the transition from laboratory-scale demonstrations to industrially viable, circular membrane systems. Future progress will depend on advancing materials design and on rethinking the entire membrane life cycle, from polymer synthesis and module fabrication to operation, recovery, and EoL management. A key challenge in creating a life cycle assessment (LCA) for membrane technologies is inconsistency with study design and reporting. This inconsistency makes it difficult to compare comparable studies. System boundaries also differ, e.g., some assess LCA as a “gate-to-gate” and do not adopt a full “cradle-to-grave” perspective, taking a complete analysis from raw material extraction, use, and end-of-life disposal. Some important phases of product life, namely decommissioning, recycling, and disposal, are often not included in the analysis, resulting in incomplete profiles of environmental impacts. Most LCA studies assessing membrane systems have a narrow focus, too. A lot of attention is aimed at environmental impact, but factors associated with sustainability, such as economic performance and social impact, are not included in the analysis. The consequences of not fully taking such factors into account will ultimately result in less complete profiles of any evaluations and less reflective of reality when decision-makers are trying to assess the sustainability of membrane technologies. In this context, the following directions highlight the most pressing needs and opportunities for the field:

1. Closed-loop design and reprocessable materials should remain a central focus of future research. Developing membranes with reversible or healable chemistries will allow multiple regeneration cycles without performance degradation, thereby reducing polymer waste and dependence on virgin feedstocks. Equally important is the advancement of green solvent systems capable of low-temperature depolymerization and integrated recovery processes that simplify recycling, minimize energy use, and enable truly sustainable membrane regeneration.
2. Module-level recycling and in situ regeneration approaches are needed to allow chemical modification and cleaning to occur directly within intact SWM modules. This includes designing controlled flow reactors or reagent circulation systems for such in situ processes to eliminate the need for destructive module disassembly and improve scalability.
3. Mechanical reliability and long-term durability must be more rigorously assessed to ensure recycled membranes can withstand real operational pressures. Standardized testing for tensile strength, compaction resistance, and fatigue behavior should be incorporated into recyclability evaluations to guarantee structural stability and lifespan. Although there is no universally accepted benchmark or minimum dataset for mechanical robustness in membrane technology, given the variation in polymer types, fabrication methods, and applications, recycled or regenerated membranes should still be tested and should demonstrate mechanical properties comparable to commercial counterparts to ensure safe and durable operation in real-world applications.
4. A comprehensive sustainability assessment should accompany every proposed recycling or upcycling approach. Detailed cost–benefit analyses, carbon footprint evaluations, and LCA are needed to validate claims of environmental advantage and identify the most impactful strategies.
5. Recycling of plastic waste for membrane fabrication should focus more on the adoption of green solvent systems, efficient solvent recovery and reuse, and standardized purification protocols to ensure material safety, performance reproducibility, and genuine sustainability in large-scale membrane manufacturing.
6. Cross-sector collaboration and policy integration are vital to bridge the gap between laboratory innovation and industrial adoption. Coordinated efforts among academia, industry, and regulatory bodies, along with incentives and certification frameworks, will be key to embedding circularity within the global membrane manufacturing sector.
7. Developing green and energy-efficient recycling schemes remains a crucial research gap. Future studies should focus on integrating green solvent systems, improving solvent recovery and reuse, and exploring cross-industry practices that can be tailored to membrane recycling technologies.

While several regeneration and recycling pathways show promise, not all are equally practical or sustainable for large-scale implementation. Solvent-intensive approaches based on CANs, or more broadly on DCPs, involve complex chemistries and require strong solvents and high temperatures, making them difficult to scale and environmentally burdensome. These methods should therefore not be prioritized for immediate industrial adoption until greener solvent alternatives are developed. In contrast, downcycling and upcycling of complete membrane modules, such as SWM modules, demonstrate strong potential for scalable application. For example, García-Pacheco et al. [48] successfully achieved whole-module recycling using a large reactor system without the need for disassembly, transforming EoL RO membranes into NF- and UF-like membranes through controlled NaClO treatment. This approach minimizes manual handling, reduces waste generation, and exemplifies a practical pathway toward circular membrane management.

In summary, while current advances have laid the scientific foundation for membrane recyclability, their translation to practice demands integrated innovation and systems engineering. With sustained interdisciplinary efforts, recycling, upcycling, and reprocessable membrane technologies could redefine the next generation of sustainable water treatment systems, helping to close the material loop in one of the most resource-intensive separation industries.

## Figures and Tables

**Table 1:** Literature summary of downcycled EoL RO membranes into functional membranes.

Original Membrane *	Recycling Procedures	Performance of Membranes	Ref.
EoL DOW^®^ Fortilife™ XC70 RO membrane.	Multi-step treatment: **Step 1**: Exposure to 0.10M NaOH solution (0 and 24 h) at 40 °C. **Step 2**: Exposure to 0.50% NaClO solution (0, 2, 4, and 6 h) at 40 °C.	The 24 h NaOH pre-treated membrane, followed by 4 h NaClO exposure, exhibited a water permeance of 27.0 LMH/bar with 62.0% Na_2_SO_4_ and 55.0% organic matter rejections, corresponding to a loose **NF-like performance**. The 6 h NaClO-treated membrane showed a much higher water permeance of ~60–90 LMH/bar with both Na_2_SO_4_ and organic matter rejections <5.00%, indicating **UF-like behavior**.	[55]
EoL TM720-400 RO membrane	Direct 8000 mg/L NaClO solution exposure at room temperature (~21.0 °C).	Treated membrane reported a stable 4.89 LMH/bar water permeance with ion rejections of 96.0%, 80.0%, 66.4%, and 74.5% for SO_4_^−2^, Cl^−1^, NO_3_^−1^, and Na^+1^, respectively, indicating an **NF-like membrane**.	[56]
EoL TMH10A-400 RO membrane	Multi-step treatment: **Step 1**: Cleaning with a mixture of 5.0 wt.% NaOH, 2.0 wt.% sodium cumene sulfonate, 5.00 wt.% EDTA, 5.0 wt.% sodium citrate dihydrate, and water. **Step 2**: Exposure to 12,385 mg/L NaClO solution for 60 h to remove the PA layer. **Step 3**: PDA coating followed by LbL self-assembly of PEI/PSS cation/anion polyelectrolyte solution.	The PEI/PSS 2.0-bilayer **NF-like membrane** showed a permeance of 7.25 LMH/bar and ions rejections of 99.0%, 99.0%, 56.5%, and 71.4% for Na_2_SO_4_, MgSO_4_, NaCl, and MgCl_2_, respectively. The PEI/PSS 1.5-bilayer NF-like membrane showed a permeance of ~9.40 ^1^ LMH/bar and ions rejections of 98.5%, 62.3%, and 86.3% and for Na_2_SO_4_, NaCl, MgCl_2_, respectively.	[57]
EoL FilmTec BW30 RO membrane	Multi-step treatment: **Step 1**: Cleaning with 0.10 wt.% NaOH followed by 0.20 wt.% HCl, each for 16 h at 25.0 °C. **Step 2**: Rewetting the membrane with 50.0% ethanol to water mixture for 5–15 min. **Step 3**: Exposure to NaOH, NaClO, H_2_O_2_, or KMnO_4_ either through immersion in the solution or recirculation at 10.0 bars.	The cleaning and rewetting slightly resorted the fouled BW30 membrane performance reaching 1.07 LMH/bar compared to 3.00 LMH/bar for pristine membrane. The 6 h 50,000 mg/L NaOH-treated membrane showed a permeance of 2.20 LMH/bar and NaCl rejection of 94.0%. ^2^ The 5.4 h 55,000 mg/L NaClO-treated membrane showed a permeance of 86.0 LMH/bar and NaCl rejection of 16.7%. ^2^ The 1 h 100,000 mg/L H_2_O_2_-treated membrane showed a permeance of 2.90 LMH/bar and NaCl rejection of 88.5%. ^3^ The 333 h 900 mg/L KMnO_4_-treated membrane showed a permeance of 45.8 LMH/bar and NaCl rejection of 25.2%. ^2^	[53]
EoL FilmTec TW30 RO membrane	Multi-step treatment: **Step 1**: Cleaning with citric acid solution (pH 2.50) for 20 min in ultrasonic bath. This was followed by cleaning with 0.20 wt.% NaOH solution at similar conditions. **Step 2**: Exposure to 110,000 mg/L NaClO solution through immersion for 2 to 5 h at room temperature (25 °C).	The EoL RO membrane showed permeance of 4.52 LMH/bar and NaCl rejection of 99.4%. The 2 h treated membrane showed a permeance of 28.8 LMH/bar and NaCl rejection of ~51.0%. ^1^ The 3 h treated membrane showed a permeance of 37.2 LMH/bar and NaCl rejection of ~30%. ^1^ The 5 h treated membrane showed a permeance of 50.4 LMH/bar and NaCl rejection of 11.6% indicating lower end UF-Like performance.	[50]
EoL BW RO membrane	Multi-step treatment: **Step 1**: Conservation in 500–1000 mg/L sodium bisulfite solution. **Step 2**: Exposure to 6000–16,000 mg/L NaClO solution by static immersion in a reactor for varying durations, without agitation.	The EoL RO membrane showed permeance of 2.07 LMH/bar and total rejection of 97.9%. The NaClO 6200 (mg/L)·hour treated membrane showed a permeance of 2.80 LMH/bar and Cl^−1,^ and Na^+1^, rejection of 76.8% and 75.4%, respectively. The NaClO 12,500 (mg/L)·hour treated membrane showed a permeance of 4.40 LMH/bar and Cl^−1^, and Na^+1^, rejection of 44.9% and 44.4%, respectively, indicated **NF-Like performance**. The NaClO 42,000 (mg/L) ·hour treated membrane showed a permeance of 30.2 LMH/bar, indicating **UF-Like performance**.	[48]
EoL FilmTec BW30-400 RO membrane	Multi-step treatment: **Step 1**: Exposure to 6250 mg/L NaClO solution for 96 h. **Step 2**: PDA/PEG co-deposition (2.00 mg/mL PEG + 2.00 mg/mL dopamine hydrochloride) for 5 and 20 h.	The 96 h 6250 mg/L NaClO treated membrane showed a permeance of 31.1 LMH/bar with ultra-low NaCl rejection and Na_2_SO_4_ rejection of 8.80% indicating **UF-like performance**. The treated and 5 h PDA/PEG-coated membrane showed a permeance of ~67.9 LMH/bar with NaCl rejection of 3.84%, Na_2_SO_4_ rejection of ~20%, and Red 23 rejection of 96.0%, indicating a **loose NF-like membrane**. The treated and 20 h PDA/PEG-coated membrane showed a permeance of ~24.0 LMH/bar with NaCl rejection of 4.10%, Na_2_SO_4_ rejection of ~20%, and Red 23 rejection of 96.4%, indicating a **loose NF-like membrane**.	[49]
EoL FilmTec BW30 RO membrane	Immersion in 75,000 (mg/L) NaClO solution for 4 h (i.e., 300,000 (mg/L)·hour contact intensity) at room temperature (25 °C)	The treated membrane showed a permeance of ~79.1 LMH/bar with NaCl rejection of 16.7%, indicating **UF-like membrane**.	[58]
EoL DOW FilmTec SW30 RO membrane	Multi-step treatment: **Step 1**: Cleaning for 24 h with 1.00 *v*/*v*% alkaline solution (EcoLab Inc. Ultrasil 110) which is a mixture of EDTA, NaOH, sodium cumene sulfonate and sodium dodecylbenzene sulfonate. **Step 2**: Exposure to 13,000–240,500 (mg/L)·hour NaClO solution through immersion under stirring conditions. **Step 3**: LbL self-assembly of cation (SC498 or F2S)/anion (SA190 or KE253) polyelectrolyte.	The 1 h 13,000 mg/L NaClO treated membrane showed a permeance of 3.70 LMH/bar with NaCl rejection of 64.0% and MgSO_4_ rejection of 76% indicating **NF-like performance**. The 18.5 h 13,000 mg/L NaClO treated membrane (240,500 (mg/L)·hour contact intensity) showed a permeance of 51.0 LMH/bar with almost zero rejections indicating **UF-like performance**. The 1 h 13,000 mg/L NaClO treated membrane and coated with eight bilayers of SC498/SA190 showed a permeance of 3.20 LMH/bar with MgSO_4_ rejection of 96.6% indicating **NF-like performance**. The >5 h and <~8 h 13,000 mg/L NaClO treated membrane and coated with eight bilayers of F2S/SA190 showed a permeance of 16.7 LMH/bar with NaCl rejection of 75.9% and MgSO_4_ rejection of 90.8% indicating **NF-like performance**	[45]

* All or selected membrane examples from each study. ^1^ Estimated from figure. ^2^ Treatment by means of immersion in the solution. ^3^ Treatment by means of solution recirculation at 10.0 bars.

**Table 2:** Summary of recent studies on recyclable membrane systems based on CANs/DCPs chemistry and key performance outcomes.

Membrane	Recycling Conditions	Performance	Ref.
Flat sheet cysteamine-crosslinked PEI (Ultem) membrane with reversible disulfide bonds.	5.00 wt./v% DTT in DMF at 60 °C; tested NMP, DMF, DCM, with DMF giving the best dissolution. Polymer recovery via 2.00 μm PTFE filtration, ethanol precipitation, and centrifugation at 12,000 rpm in three cycles of 20.0 min. Membrane refabrication following the NIPS method.	Recycled PEI membrane permeance~6.20 LMH/bar. Crosslinked PEI membrane permeance~1.12 LMH/bar. Recycled membrane DR80 dye rejection~85.0% ^1^ compared to~99.0% in crosslinked PEI membranes.	[83]
Flat sheet furan-functionalized oligomer (FGE-D230) crosslinked with BMI in the presence of DMF and PEG.	Thermal depolymerization at ~140 °C in DMF to trigger retro-DA reaction. Filtration using filter paper, and liquid extraction in DCM solvent to recover oligomers. Membrane refabrication following the NIPS method.	Recycled membrane permeance~24.9 LMH/bar ^1^. Original membrane permeance~28.5 LMH/bar. Original and recycled membrane dye rejection ~ 80.0% ^1^.	[82]
Electrospun Copolymer poly[(furfuryl methacrylate)-co-(butyl methacrylate)] (FMA-co-HFBMA) membrane with BMI.	Thermal depolymerization at ~140 °C in DMF to trigger retro-DA decrosslinking reaction. Filtration using filter paper to remove oil and large particles from the solution to obtain a clean polymer solution. Membrane refabrication following the electrospinning method.	Recycled membrane flux is slightly lower than the original membrane. Both original and recycled membranes showed comparable oil/water separation efficiency.	[85]
Flat sheet furfurylamine-modified poly(amide-imide) (PAI-FU) membrane crosslinked with BMI in acetone.	Thermal depolymerization at ~120 °C in DMF to trigger retro-DA de-crosslinking reaction. **Approach 1**: The solution was precipitated in water, and the recovered polymer was washed with acetone and dried for reuse in NIPS membrane fabrication. **Approach 2**: The solution was directly cast on a Teflon dish and evaporated under nitrogen for 5 days, then washed and vacuum-dried to form a dense recycled membrane.	Original PAI-FU-BMI membrane acetone permeance~3.68 LMH/bar. Original PAI-FU-BMI Rose Bengal dye rejection ~ 95.5%. Recycled membranes **via approach 1** were brittle and contained major cracks, thus performance testing was not possible. The recycled membrane **via approach 2** was dense and non-porous, thus performance testing was not possible.	[86]
Flat sheet PVDF membrane with dopamine-anchored BMI-GO.	Thermal depolymerization at ~140 °C in DMF to trigger retro-DA de-crosslinking reaction. Liquid extraction using DCM solvent and water to produce two phases, an aqueous phase containing foulants and an organic phase with the polymer and DA precursors. The foulants were removed while the polymeric fraction was recovered by solvent evaporation and refabricated into new membranes using the NIPS method.	Recycled membrane flux ~ 285 LMH ^1^ at 100 psi. Original membrane flux ~ 275 LMH at 100 psi. Original membrane dye rejection ≥97.0%. Recycled membrane dye rejection~94.0% (Congo Red) and 87.0% (Methylene Blue).	[87]
Electrospun Poly(FMA-co-BMA) (PFB) membrane with BMI.	Thermal depolymerization at ~140 °C in DMF to trigger retro-DA de-crosslinking reaction. Filtration using filter paper to remove oil and large particles from the solution to obtain clean polymer solution. Membrane refabrication following electrospinning method.	Original membrane oil–water emulsion flux~647 LMH. Recycled membrane oil–water emulsion flux~824 LMH (3rd recycled membrane). Original membrane oil–water separation efficiency ~98.8%. Recycled membrane oil–water separation efficiency ~97.0% (3rd recycled membrane).	[88]

^1^ Estimated from the figure, D230: polyetheramine D-230, FGE: furfuryl glycidyl ether, DA: Diels–Alder, GO: graphene oxide.

## Data Availability

No data were used in this study.
